# Platypnea Orthodeoxia Syndrome Secondary to a Persistent Eustachian Valve

**DOI:** 10.7759/cureus.42900

**Published:** 2023-08-03

**Authors:** Poonam Rao, Chaitanya Undavalli, Mohamed Ghoweba, Aparna Kanuparthy, Khashayar Vahdat

**Affiliations:** 1 Internal Medicine, CHRISTUS Health/Texas A&M School of Medicine Internal Medicine Residency Program, Longview, USA; 2 Internal Medicine, Texas A&M College of Medicine/CHRISTUS Good Shepherd Medical Center, Longview, USA; 3 Cardiology, CHRISTUS Health/Texas A&M School of Medicine Internal Medicine Residency Program, Longview, USA

**Keywords:** patent foramen ovale (pfo), elongated aorta, right-to-left shunting, thoracic kyphosis, persistent eustachian valve, platypnea-orthodeoxia syndrome

## Abstract

Platypnea-Orthodeoxia syndrome (POS) is a rare and poorly understood syndrome characterized by platypnea and oxygen desaturation in the upright position that is relieved by recumbency. Here, we report a case of an 84-year-old woman who had chronic hypoxia in an upright position despite using home oxygen. The patient presented for hypoxia evaluation and was noted to have a restrictive pattern on pulmonary function tests (PFT). An echocardiogram showed a prominent eustachian valve extending from inferior to superior vena cava with contrast approaching the interatrial septum. The patient had a complete resolution of her platypnea following the closure of the patent foramen ovale.

## Introduction

Platypnea-Orthodeoxia syndrome (POS) was first described in the late 1940s; however, the underlying pathophysiologic triggers are still not fully understood. Two major components believed to play a role in this physiology are anatomical and functional components. The anatomical component is responsible for shunting from the right to the left side with or without increased right-sided cardiac pressures. Shunting etiology could be intracardiac (patent foramen ovale (PFO), atrial septal defect (ASD)), pulmonary (hepatopulmonary, pulmonary arteriovenous malformations), a ventilation-perfusion mismatch, or a combination of these. Eustachian valve forms commonly associated with POS have associated PFOs and ASDs. They occur in patients with prominent valves and ASDs without pulmonary hypertension or right ventricular outflow obstruction [1-3]. The functional component promotes shunting when the patient rises to an upright position. We report our case of chronic hypoxemia in an 84-year-old female with kyphosis. She was evaluated multiple times for her hypoxia in the outpatient settings and was diagnosed with restrictive lung disease based on PFTs and was subsequently put on home oxygen with little benefit.

## Case presentation

An 84-year-old woman with a medical history significant for chronic hypoxic respiratory failure on 2-3 L of home oxygen by nasal cannula (unclear etiology), coronary artery disease with six prior myocardial infarctions (most recent myocardial infarction 11 years ago), atrial flutter, hypertension, hypothyroidism, type 2 diabetes mellitus controlled with oral medications, and class II obesity presented to the emergency department with worsening shortness of breath and fatigue, particularly on exertion. The patient was admitted to the intensive care unit to evaluate and manage her hypoxic respiratory failure. Cardiology was consulted to evaluate several months of dyspnea at rest and with exertion, palpitations, fatigue, intermittent chest pain, and desaturations despite 2-3 L of oxygen supplementation via nasal cannula at home. Her dyspnea was reported to have worsened while upright and alleviated on lying down. Patient was on aspirin, atorvastatin, metoprolol, lisinopril, amiodarone, and dabigatran. 

On admission, vitals were unremarkable except for an elevated blood pressure of 162/94 mmHg. Physical exam was significant for kyphosis, decreased breath sounds bilaterally, a loud P2 sound on auscultation, and 1+ pitting edema on the lower extremities bilaterally. A 6-minute walk test in the hospital demonstrated oxygen saturation in the mid-80s. However, the patient was saturating at 86% in the supine position on 4 L nasal cannula, the arterial blood gas was significant for hypoxia (PO_2_ 77%) with a normal partial pressure of carbon dioxide (PCO_2_ 36), and hence the patient remained on oxygen supplementation to raise her oxygen saturation above 94%. Chest X-ray demonstrated mild cardiac enlargement without pulmonary edema. Pulmonary function tests (PFTs) revealed a forced expiratory volume in 1 second to forced vital capacity ratio (FEV1/FVC) of 0.72, a total lung capacity (TLC) of 109% of predicted, and a diffusing capacity for carbon monoxide (DLCO) of 67%. 

**Figure 1:**
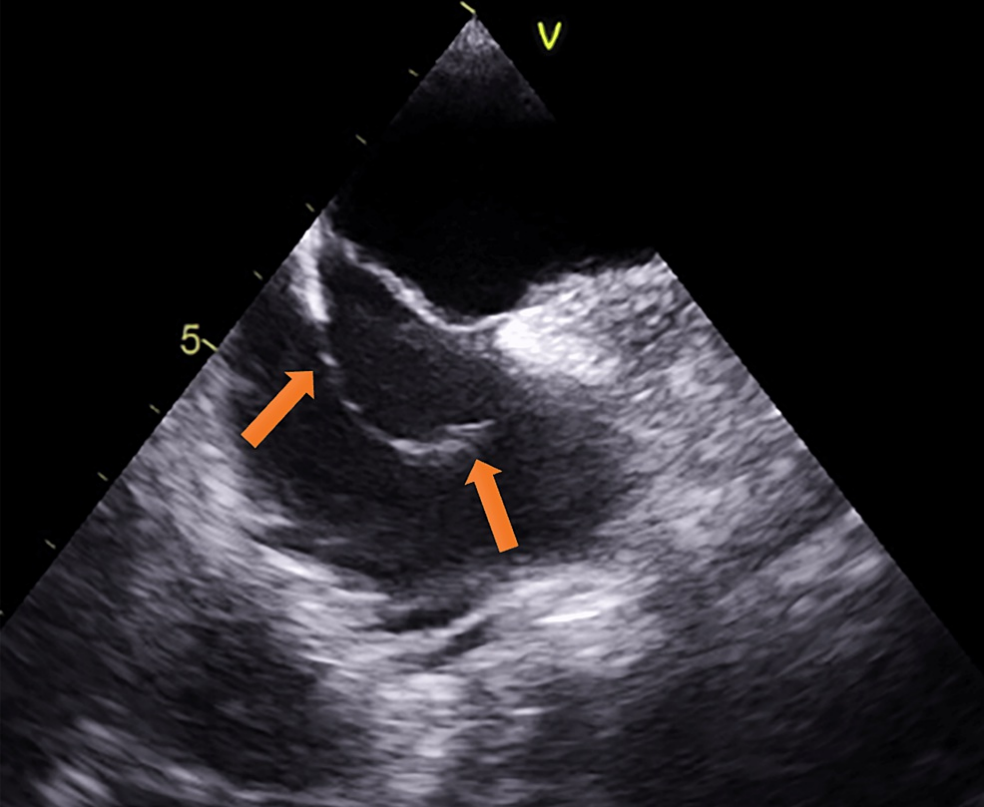
A TEE demonstrating a persistent eustachian valve extending from the inferior vena cava to the superior vena cava (arrows). TEE: Transesophageal Echocardiogram

A transthoracic echocardiogram (TTE) with agitated saline contrast study showed shunting from the right atrium to the left atrium concerning PFO/ASD and a normal RVSP. A transesophageal echocardiogram (TEE) revealed a normal ventricular size, thickness, and function. The right and left atria were normal in size, with an aneurysmal interatrial septum with a right to left shunting. A prominent eustachian valve was identified extending from the inferior vena cava (IVC) to the superior vena cava (SVC) that diverted contrast to the interatrial septum/PFO (Video 1, Figure 1).

**Video 1:** A TEE showing a persistent eustachian valve extending from the inferior vena cava to the superior vena cava. TEE: Transesophageal Echocardiogram

 

**Figure 2:**
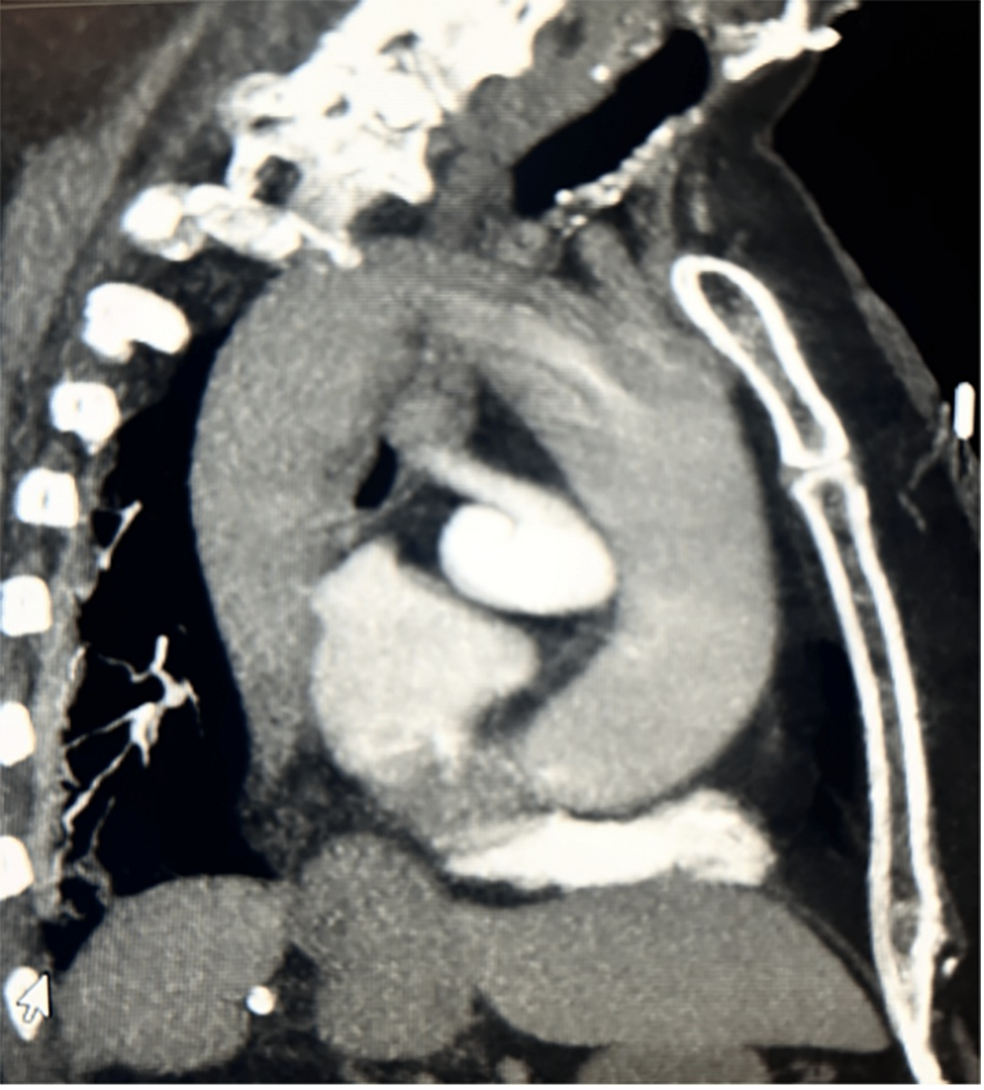
A CT chest with contrast scan demonstrates an elongated aorta. CT: Computed Tomography

Cardiac catheterization did not show significant macrovascular coronary artery disease. Cardiac magnetic resonance imaging (MRI) was unremarkable. A computed tomography (CT) scan of the chest performed to rule out congenital anomalies demonstrated an elongated aorta and an incidental small subsegmental pulmonary embolism not contributing to the patient dyspnea at the time of admission; hence, the patient was started on anticoagulation with apixaban 5 mg twice daily. Formal upright (SpO_2_ 86%) and supine oxygen saturations (SpO_2_ 94%) were recorded to diagnose the patient with POS (Figure 2, Video 2).

 

**Video 2:** TEE pre- and post-PFO closure TEE: Transesophageal echocardiogram, PFO: patent foramen ovale

Upon shared decision-making with the patient, she underwent a successful PFO closure with an AmplatzerTM Septal Occluder with complete resolution of her hypoxia with oxygen saturations above 92% on room air. The patient could walk, sit, and talk without distress and did not require supplemental oxygen. After discharge from the cardiac rehabilitation unit, the patient regained a significant proportion of her activities of daily living (ADLs).

## Discussion

The eustachian valve is an embryologic remnant of the inferior vena cava (IVC) valve at the junction of the IVC and inferior right atrium (RA). In fetal development, the valve directs incoming oxygenated blood toward the foramen ovale and away from the tricuspid valve. The eustachian valve typically has no physiological significance after the closure of the foramen ovale and remains non-functional in normal adults.  The embryologic remnant can present differently on echocardiography, ranging in thickness, length, and shape. While the valve is usually benign, veno-arterial shunting is a rare phenomenon described in a few case reports [1-3]. The increase in the right atrial pressure increases shunting, but in patients with persistent eustachian valves, shunting can occur despite the normal right atrial pressure through the PFO that is positional in nature [4].

A persistent eustachian valve can have variable clinical presentations. Normally, the valve completely disappears or remains as a thin rim. The most common presentation in adults is a crescentic fold of the endocardium arising from the anterior rim visualized on echocardiography. Rarely can the valve persist as an elongated structure projecting into the RA, demonstrating an undulating motion on echocardiographic imaging [5]. A persistent eustachian valve rarely presents with clinical manifestations in the absence of structural heart disease; hence, such infants can be monitored conservatively [6]. In contrast, large persistent eustachian valves have been reported to increase the right to left shunting in patients with structural heart diseases like ASD/PFO by directing the blood flow through the defect into the left atrium. These patients present with cyanosis during early infancy and are successfully treated with the surgical repair of the defect [2,7].

Different aortic pathologies, including aortic aneurysms [8], aortic root dilations [9], and elongation of the aorta, especially in patients with atrial septal aneurysm [10], persistent eustachian valve, or a Chiari’s Network can be seen in patients with intracardiac POS [11]. The presence of these aortic anatomical abnormalities that progress with aging, along with associated exacerbating factors like diaphragmatic paralysis or restrictive lung etiologies, plays an important role in the late presentation of POS in adults.

With growing age, the flow of the remnant eustachian valve is redirected to the PFO. Patients with kyphosis, and spinal shortening like our patient, are assumed to possess an altered intrathoracic pressure and form an elongated aorta which aids in the shunting [12]. While the patient is upright, the PFO diverts more blood flow to the LA [13].

Treatment mainly entails the closure of the PFO by implantation of a septal occluding device. Most patients have a dramatic improvement in their symptoms. Closure of the defect also reduces the risk of future stroke [10]. Mojadidi et al. studied the effects of PFO closure with POS [13]. They found that approximately 65% of patients had significant improvement in oxygen saturation after the closure procedure, with some patients even having complete resolution of their dyspnea and hypoxemia. Patients with no signs of improvement following PFO closure were identified to have a pulmonary cause underlying their hypoxia [14].

## Conclusions

POS should be part of the differentials list while evaluating patients with hypoxia. It is a complex diagnosis demanding a high degree of clinical suspicion. Patients can present late in their seventh or eighth decade due to slow progression. A persistent eustachian valve in a setting of kyphosis with an elongated aorta can lead to POS. The valve shunts more blood through the PFO in the upright position leading to a more pronounced hypoxia. Patients presenting with signs and symptoms of POS should undergo a complete cardiac and pulmonary workup.
